# Assessment of serum caveolin-3 levels in patients with heart failure

**DOI:** 10.3389/fcvm.2025.1630737

**Published:** 2025-10-10

**Authors:** Ahmet Süsenbük, Hakan Kaya, Sabri Abuş, Abdulmecit Afşin

**Affiliations:** ^1^ Department of Cardiology, Abdülkadir Yüksel State Hospital, Gaziantep, Türkiye; ^2^ Department of Cardiology, Adıyaman University, Adıyaman, Türkiye

**Keywords:** heart failure, caveolin-3, left ventricular ejection fraction, NT-ProBNP, biomolecules

## Abstract

**Background:**

Heart failure (HF) is a complex syndrome caused by structural and functional abnormalities that impair ventricular filling and ejection. Caveolin-3 (Cav-3), a muscle-specific membrane protein, is essential for T-tubule formation and maintenance in cardiomyocytes. Although caveolin deficiency leads to severe cardiac phenotypes, Cav-3's specific mechanistic role in chronic HF remains insufficiently defined.

**Objective:**

The principal objective of this investigation was to assess serum Cav-3 concentrations in patients diagnosed with chronic HF.

**Methods:**

This case-control study encompassed 90 participants, comprising 45 individuals with chronic HF (HF group) and 45 age- and sex-matched healthy controls (non-HF group). Blood specimens were obtained from both groups, and Cav-3 concentrations were quantified utilizing the enzyme-linked immunosorbent assay (ELISA) methodology. In addition, all participants underwent comprehensive transthoracic echocardiography (TTE). Both echocardiographic and laboratory parameters, including Cav-3 levels, were systematically compared between the two cohorts.

**Results:**

Among 90 participants (45 HF; 45 matched controls), HF patients showed typical adverse remodeling [LVEF 35% [20–50] vs. 60% [55–65], *p* < 0.001] and higher inflammatory/coagulation activity. Median serum Cav-3 was higher in HF than controls [4.83 [4.34–5.60] vs. 3.97 [3.30–4.96] ng/L; *p* < 0.001]. On ROC analysis, NT-proBNP provided the strongest single-marker discrimination (AUC 0.850; cutoff 254.50 pg/ml; sensitivity 79.5%; specificity 80.0%), Cav-3 alone showed moderate accuracy (AUC 0.705; cutoff 4.36 ng/L; sensitivity 75.0%; specificity 73.3%), and the Cav-3 + NT-proBNP combination achieved the highest AUC (0.878; sensitivity 81.8%; specificity 84.4%; *p* < 0.001). In multivariable models predicting EF, WBC, NT-proBNP, and ESR were independent negative predictors, whereas Cav-3 was not significant after adjustment. Cav-3 concentrations were higher in HFrEF and HFmrEF vs. controls, with no difference between HF subgroups.

**Conclusions:**

Serum Cav-3 is elevated in chronic HF and enhances diagnostic discrimination when added to NT-proBNP, but does not independently predict EF after adjustment. These findings support Cav-3 as an adjunctive—rather than stand-alone—biomarker within a multimarker strategy. Prospective multicenter studies should validate reproducibility, define clinically actionable thresholds, and quantify incremental value over natriuretic peptide–based and multimarker baselines.

## Introduction

Heart failure (HF) remains a high-burden syndrome in which natriuretic peptides (BNP/NT-proBNP) are foundational for diagnosis and risk stratification, yet their interpretation is frequently confounded by comorbidities—most notably obesity, atrial fibrillation, and chronic kidney disease—highlighting the need for complementary, pathophysiology-anchored biomarkers (1).

Caveolin-3 (Cav-3) is the muscle-specific scaffold of caveolae within cardiomyocyte T-tubules that organizes β-adrenergic/L-type Ca^2+^ channel signaling microdomains essential for excitation–contraction coupling. In hypertrophy and HF, Cav-3 expression falls with T-tubule remodeling, and loss-of-function or disorganization of Cav-3 disrupts these signaling hubs. Conversely, cardiomyocyte-targeted Cav-3 overexpression preserves T-tubule structure and dampens pathological signaling, positioning Cav-3 at a mechanistic intersection of structural remodeling and impaired signaling in HF (2, 3).

Multiple preclinical lines of evidence suggest that augmenting or stabilizing Cav-3 could be therapeutically beneficial in HF. Cardiac myocyte–specific Cav-3 overexpression attenuates hypertrophy, modulates pathological Ca^2+^/calcineurin–NFAT signaling, and enhances natriuretic peptide expression (4, 5). Cav-3 overexpression preserves T-tubule architecture and excitation–contraction coupling during pressure overload or failure, a key substrate for systolic dysfunction reversal (3). Cav-3-dependent caveolae mediate ischemic preconditioning; Cav-3 upregulation can mimic this protection, supporting a causal, druggable role of Cav-3 microdomains (6). Cardiac AAV9 vectors have been used to manipulate Cav-3 signaling *in vivo* and have advanced microdomain-restoring strategies (e.g., cBIN1) toward translation, underscoring the deliverability of membrane-microdomain targets in large-animal and disease models (7–9). Reviews highlight caveolins (including Cav-3) as tractable drug targets; caveolin scaffolding–domain–based strategies and peptide modulators provide additional proof-of-concept for non-genetic therapeutics aimed at caveolar signaling (10, 11).

Despite this biological and translational plausibility, circulating (serum) Cav-3 has scarcely been evaluated in humans. A small study in atrial fibrillation suggested associations between serum Cav-3 and atrial/clinical remodeling, but its diagnostic or prognostic value in chronic HF—and its relationship to natriuretic peptides and echocardiographic indices—remains undefined (12).

Accordingly, we aimed to (i) compare serum Cav-3 levels in patients with chronic HF vs. matched controls and (ii) examine associations between Cav-3 and NT-proBNP as well as echocardiographic measures of structure and function. We hypothesized that serum Cav-3 would differ between HF and controls and would track adverse remodeling independently of natriuretic peptides.

## Methods

### Study design and sample

Ethical approval for the execution of this investigation was duly granted by the Ethics Committee for Non-Interventional Clinical Research at the Faculty of Medicine, Adıyaman University (IRB Number: 2021/01–26). The study was meticulously conducted in strict adherence to the principles delineated in the Declaration of Helsinki, as well as in conformity with the Good Clinical Practice (GCP) guidelines. Informed consent, duly written and signed by each participant, was obtained prior to their inclusion in the study, ensuring full compliance with ethical standards.

This investigation was designed as a case-control study. All individuals presenting consecutively to the cardiology outpatient clinic at Adıyaman Training and Research Hospital between the period of 01/02/2021 and 01/09/2021 were subjected to a rigorous evaluation to determine their eligibility for inclusion in the study. The patient cohort (*n* = 45) comprised individuals diagnosed with chronic HF aged between 18 and 80 years. In parallel, the control group (*n* = 45) consisted of age- and sex-matched individuals who exhibited no clinical signs or diagnostic evidence of HF.

### Inclusion and exclusion criteria

Patients aged 18 years or older, diagnosed with chronic HF and exhibiting a left ventricular ejection fraction (LVEF) of less than 50%, in accordance with the prevailing guidelines for HF, were incorporated into the study. Those presenting with an LVEF ≤40% were classified under the category of HF with reduced ejection fraction (HFrEF), while individuals with an LVEF ranging from 41% to 49% were designated as having HF with mildly reduced ejection fraction (HFmrEF). The control group was comprised of individuals exhibiting an LVEF greater than 50%, without any clinical evidence or symptomatic manifestations indicative of HF.

Exclusion criteria encompassed individuals with decompensated HF, HF with preserved ejection fraction, AF, acute renal failure, active sepsis, pregnant women, acute coronary syndrome, acute cerebrovascular accidents (CVA), end-stage renal disease, or liver failure.

### Echocardiographic assessment

Both the HF patients and control subjects underwent transthoracic echocardiography (TTE). This diagnostic procedure was meticulously conducted for both cohorts in the left lateral decubitus position after an interval of five minutes of rest, utilizing the advanced Philips EPIQ 7 ultrasound system, in strict accordance with the contemporary standards outlined by the American Society of Echocardiography. Standardized echocardiographic imaging protocols were employed to ascertain critical parameters, including LVEF, left ventricular end-systolic diameter (LVESD), end-diastolic diameter (LVEDD), left atrial diameter (LAD), Tricuspid Annular Plane Systolic Excursion (TAPSE), the thickness of the interventricular septum (IVS), and left ventricular posterior wall (PW), in addition to Doppler-derived echocardiographic images. To ensure continuous cardiac monitoring and minimize procedural risks, all participants were simultaneously observed through electrocardiographic telemetry throughout the course of the procedure.

### Measurement of Cav-3 and other blood parameters

A single 10 ml venous blood specimen was meticulously collected from the antecubital vein of each participant. Subsequently, the samples underwent centrifugation at a precise 4,000 rpm for a duration of 10 minutes within EDTA-coated tubes, after which the resultant serum was carefully isolated and preserved in Eppendorf tubes at a sub-zero temperature of −80°C for long-term storage. Notably, the blood samples were subjected to a singular thawing cycle prior to any analytical measurements. The concentrations of Cav-3 were determined utilizing a specialized Human Caveolin-3 ELISA kit, adhering to the enzyme-linked immunosorbent assay (ELISA) methodology. Concurrently, additional blood parameters, including serum glucose, NT-proBNP, and D-dimer levels, were systematically analyzed according to rigorously standardized protocols established for such assessments.

### Statistical analysis

The data underwent comprehensive statistical analysis utilizing SPSS version 26.0 for Macintosh (IBM Corp., Armonk, NY). The distribution of continuous variables was meticulously evaluated for normality using the Kolmogorov–Smirnov test. Categorical data were expressed as frequencies (n) and percentages (%). For continuous variables that conformed to a normal distribution, results were presented as the mean ± standard deviation, while for those exhibiting a non-normal distribution, median values with 25. and 75. interquartile ranges (IQR) were presented. In the context of pairwise comparisons, an independent samples *t*-test was utilized for variables conforming to normal distribution, whereas Pearson's chi-squared test was employed for those variables that did not conform to normality. To evaluate the diagnostic performance of various tests, including sensitivity and specificity, Receiver Operating Characteristic (ROC) analysis was meticulously conducted, and the resultant ROC curves were subsequently plotted, highlighting the significant differences observed between the groups under investigation. Linear regression was performed to assess the effects of the independent variables on EF.

## Results

### Baseline characteristics

As shown in Table 1, the groups were comparable in age (66.40 ± 10.18 vs. 63.87 ± 9.76 years; *p* = 0.465) and smoking status (33.3% vs. 28.9%; *p* = 0.649). Sex distribution trended toward more males in controls (57.8% vs. 37.8%) but did not reach significance (*p* = 0.058).

**Table 1:** Comparison of sociodemographic characteristics and comorbidities between HF patients and non-HF individuals.

Parameter	HF (*n* = 45)	Non-HF (*n* = 45)	*p*-value
Age (years)	66.40 ± 10.18	63.87 ± 9.76	0.465^a^
Sex			0.058^b^
Female	28 (62.2)	19 (42.2)	
Male	17 (37.8)	26 (57.8)	
DM	18 (40.0)	5 (11.1)	**0**.**002**^b^
HT	34 (75.6)	18 (40.0)	**0**.**001**^b^
CAD	40 (88.9)	16 (35.6)	**0**.**001**^b^
CVA	2 (4.4)	0 (0)	0.494
Smokers	15 (33.3)	13 (28.9)	0.649^b^

Data are presented as *n* (%) or mean ± SD.

Bold indicates statistically significant values.

CAD, coronary artery disease; CVA, cerebrovascular accidents; DM, diabetes mellitus; HF, heart failure; HT, hypertension; SD, standard deviation.

^a^ Student's *t*-test was used.

^b^ Chi-square test was used.

Metabolic–cardiovascular comorbidities were substantially more prevalent in HF: DM 40.0% vs. 11.1% (*p* = 0.0022), HT 75.6% vs. 40.0% (*p* = 0.0012), and CAD 88.9% vs. 35.6% (*p* = 0.0012). Prior CVA was rare and similar (4.4% vs. 0%; *p* = 0.494). These patterns confirm a heavier comorbidity burden in the HF cohort while baseline demographics remain broadly comparable.

### Echocardiographic parameters

Echocardiography findings (Table 2) reflected typical HF remodeling. Median LVEF was markedly lower in HF [35% (20–50)] than in non-HF [60% (55–65); *p* < 0.001]. Left-sided chamber dimensions were larger in HF: LVESD 43.58 ± 8.04 mm vs. 35.69 ± 4.03 mm and LVEDD 53.02 ± 8.34 mm vs. 46.42 ± 3.74 mm (both *p* < 0.001). LAD was greater (42.56 ± 6.47 mm vs. 33.24 ± 3.87 mm; *p* < 0.001) and wall thicknesses were increased (IVS median 12 vs. 11 mm, *p* = 0.021; PW median 11 vs. 8 mm, *p* < 0.001). Right-sided function was reduced: TAPSE 16.87 ± 3.48 mm vs. 26.00 ± 3.67 mm (*p* < 0.001). PAP (listed as PAB in Table 2) was higher in HF (median 25 vs. 16 mmHg; *p* < 0.001), whereas IVC diameter did not differ significantly (15.42 ± 2.71 vs. 14.67 ± 2.21 mm; *p* = 0.152). Overall, HF patients showed a consistent pattern of adverse remodeling and impaired systolic function.

**Table 2:** Comparison of echocardiographic measurements between HF patients and Non-HF individuals.

Parameter	HF (*n* = 45)	Non-HF (*n* = 45)	*p*-value
LVEF (%)	35 (20–50)	60 (55–65)	**<0**.**001**^a^
LVESD (mm)	43.58 ± 8.04	35.69 ± 4.03	**<0**.**001**^b^
LVEDD (mm)	53.02 ± 8.34	46.42 ± 3.74	**<0**.**001**^b^
LAD (mm)	42.56 ± 6.47	33.24 ± 3.87	**<0**.**001**^b^
IVS diameter (mm)	12 (11–13.5)	11 (10–12)	**0**.**021**^a^
PW thickness (mm)	11 (9.5–12.5)	8 (8–9.5)	**<0**.**001**^a^
IVC diameter (mm)	15.42 ± 2.71	14.67 ± 2.21	0.152^b^
PAB (mmHg)	25 (0–47.5)	16 (0–10)	**<0**.**001**^a^
TAPSE (mm)	16.87 ± 3.48	26.00 ± 3.67	**<0**.**001**^b^

Data are presented as mean ± SD, or median (min-max).

Bold indicates statistically significant values.

HF, heart failure; IVC, inferior vena cava; IVS, interventricular septum; LAD, left atrial diameter; LVEF, left ventricular ejection fraction; LVESD, left ventricular end-systolic diameter; LVEDD, left ventricular end-diastolic diameter; LAD, left atrial diameter; PAP, pulmonary arterial pressure; PW, posterior wall; TAPSE, tricuspid annular plane systolic excursion.

^a^ Mann–Whitney *U*-test was used.

^b^ Student's *t*-test was used.

### Laboratory findings and serum Cav-3 distribution

Between-group laboratory comparisons (Table 3) showed higher inflammation and neurohormonal activation in HF. Specifically, WBC was higher (7.94 ± 2.23 vs. 6.61 ± 2.37 × 10³/µl; *p* = 0.008), ESR was elevated (13.20 ± 9.46 vs. 5.24 ± 6.04 mm/h; *p* < 0.001), and NT-proBNP was markedly increased (median 705 vs. 103 pg/ml; *p* < 0.001). D-dimer was higher (median 504 vs. 348 ng/ml; *p* = 0.001), and potassium was modestly higher (4.54 ± 0.42 vs. 4.26 ± 0.35 mEq/L; *p* = 0.001).

**Table 3:** Comparison of laboratory parameters between HF patients and Non-HF individuals.

Parameter	HF (*n* = 45)	Non-HF (*n* = 45)	*p*-value
Hemoglobin, mg/dl	12.76 ± 2.37	12.98 ± 1.48	0.607^a^
WBC, 10^3^/μL	7.94 ± 2.23	6.61 ± 2.37	**0**.**008**^a^
Platelets, 10^3^/μL	251.02 ± 77.56	225.84 ± 55.42	0.080^a^
Glucose, mg/dl	129 (99–176.5)	94 (88–156)	**<0**.**001**^b^
Creatinine, mg/dl	0.75 (0.90–1.10)	0.67 (0.51–1.06)	**<0**.**001**^b^
Urea, mg/dl	38 (27–59.5)	34 (25–54)	0.157^b^
Na, mEq/L	136.42 ± 3.26	134.82 ± 17.89	0.557^a^
K, mEq/L	4.54 ± 0.42	4.26 ± 0.35	**0**.**001**^a^
Troponin I, ng/ml	0.01 ± 0.01	0.01 ± 0.02	0.945^a^
NT-proBNP, pg/ml	705 (291.5–2,690)	103 (70–246)	**<0**.**001**^b^
D-dimer, ng/ml	504 (361–1,520)	348 (240–457)	**0**.**001**^b^
CRP, mg/dl	0.30 (0.10–5.30)	0.30 (0.10–2.00)	0.166^b^
PCT, ng/ml	0.12 (0.12–0.41)	0.12 (0.12–0.40)	0.993^b^
ESR, mm/h	13.20 ± 9.46	5.24 ± 6.04	**<0**.**001**^a^
Cav-3, ng/L	4.83 (4.34–5.60)	3.97 (3.30–4.96)	**<0**.**001**^b^

Data are presented as mean ± SD, or median (min-max).

Bold indicates statistically significant values.

Cav-3, caveolin-3; CRP, C-reactive protein; ESR, erythrocyte sedimentation rate; K, potassium; Na, sodium; NT-proBNP, N-terminal pro-brain natriuretic peptide; PCT, procalcitonin; WBC, white blood cells.

^a^ Student's *t*-test was used.

^b^ Mann–Whitney *U*-test was used.

Renal indices showed higher creatinine in HF (*p* < 0.001), while urea differences were non-significant (*p* = 0.157). Hemoglobin, platelets, sodium, troponin-I, CRP, and PCT did not differ (all *p* > 0.05).

**Figure 1:**
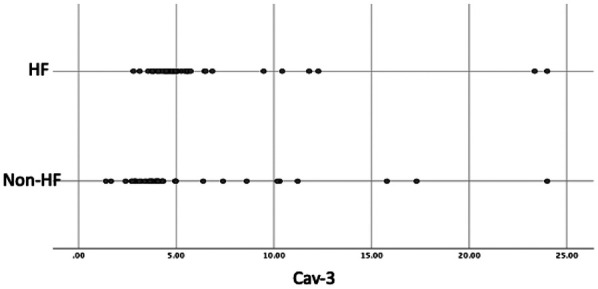
Dot Plot of Cav-3 Values in the Groups*.* Cav-3, caveolin-3; HF, heart failure.

Importantly, serum Cav-3 was higher in HF [median 4.83 (4.34–5.60) ng/L] than in non-HF [3.97 (3.30–4.96) ng/L; *p* < 0.001]. Groupwise distributions are shown in Figure 1.

### Diagnostic performance (ROC analysis)

**Figure 2:**
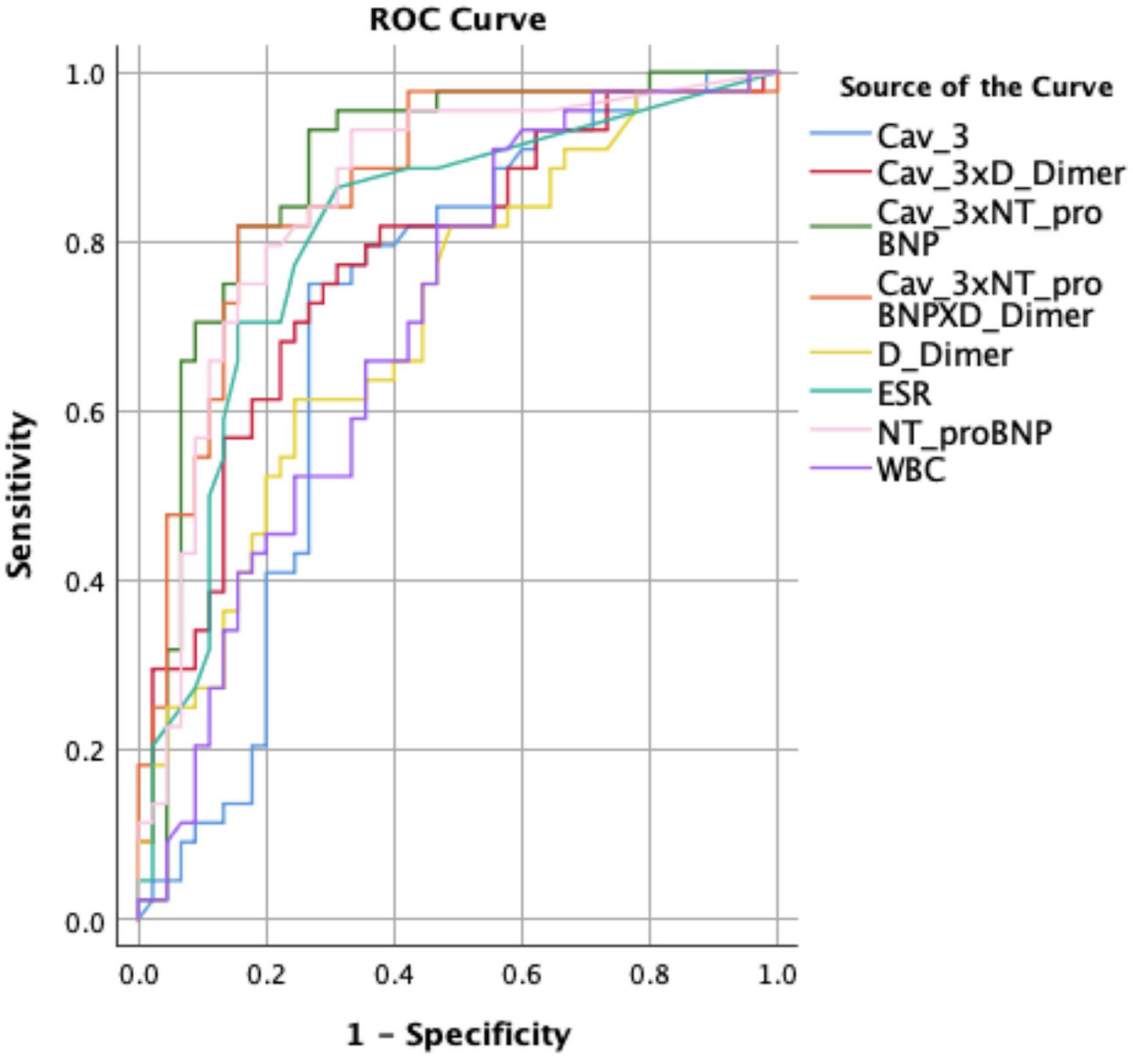
ROC curve of the parameters showing differences between the groups*.* Cav-3, caveolin-3; ESR, erythrocyte sedimentation rate; NT-proBNP, N-terminal pro-brain natriuretic peptide; WBC, white blood cells.

ROC analyses for variables with significant between-group differences are summarized in Table 4 and Figure 2. Individually, NT-proBNP provided the highest discrimination (AUC = 0.850; cutoff 254.50 pg/ml; sensitivity 79.5%; specificity 80.0%). ESR and D-dimer each showed good/moderate discrimination (AUC = 0.807 and 0.709, respectively), while Cav-3 alone yielded a moderate AUC (0.705; cutoff 4.36 ng/L; sensitivity 75.0%; specificity 73.3%). WBC showed lower discrimination (AUC = 0.700).

**Table 4:** ROC analysis of the parameters showing differences between groups.

Parameter	AUC	Cut-off	Sensitivity	Specificity	*p*-value
WBC	0,700	7.05	65.9%	64.4%	**0**.**001**
NT-proBNP	0.850	254.50	79.5%	80.0%	**<0**.**001**
D-dimer	0.709	399.00	63.6%	64.4%	**0**.**001**
ESR	0.807	5.50	77.3%	75.6%	**<0**.**001**
Cav-3	0.705	4.36	75.0%	73.3%	**0**.**001**
Cav-3* NT-proBNP	0.878	1,245.75	81.8%	84.4%	**<0**.**001**
Cav-3* D-dimer	0.774	1,966.51	75.0%	71.1%	**<0**.**001**
Cav-3* NT-proBNP* D-dimer	0.863	550,619.45	81.8%	84.4%	**<0**.**001**

AUC, area under the ROC curve; LR, likelihood ratio; Cav-3, caveolin-3; ESR, erythrocyte sedimentation rate; NT-proBNP, N-terminal pro-brain natriuretic peptide; WBC, white blood cells.

Crucially, biomarker combinations improved classification: Cav-3 + NT-proBNP achieved the best overall performance (AUC = 0.878; sensitivity 81.8%; specificity 84.4%; *p* < 0.001). Cav-3 + D-dimer also outperformed either marker alone (AUC = 0.774), and the triple combination (Cav-3 + NT-proBNP + D-dimer) remained high (AUC = 0.863). These results indicate that Cav-3 contributes additive discrimination when paired with established biomarkers, particularly NT-proBNP.

### Multivariable prediction of ejection fraction

In a linear regression including WBC, NT-proBNP, D-dimer, ESR, and Cav-3 (Table 5), the model was significant [F(5,83) = 8.65, *p* < 0.001] and explained 30% of EF variance (adjusted R² = 0.303). Independent negative predictors of EF were:

• WBC (B = −1.705, *p* = 0.004; 95% CI −2.847 to −0.564) — roughly 1.7 percentage-point lower EF per 1 × 10³/µl increase.
• NT-proBNP (B = −0.001, *p* = 0.012; 95% CI as in Table 5) — approximately 0.1 percentage-point lower EF per 100 pg/ml increase.
• ESR (B = −0.594, *p* = 0.001; 95% CI −0.920 to −0.268) — about 0.6 percentage-point lower EF per 1 mm/h increase.
• D-dimer and Cav-3 were not significant after adjustment (*p* = 0.442 and *p* = 0.227, respectively). The intercept was 69.37 (*p* < 0.001).

**Table 5:** Predictors of ejection fraction (EF) identified by linear regression analysis.

Parameter	B	Beta	p-m	%95 confidence interval	
				Lower bound	Upper bound
WBC	−1.705	−0.270	0.004	−2.847	−0.564
NT-proBNP	−0.001	−0.235	0.012	−0.002	<0.001
D-dimer	<0.001	−0.074	0.442	−0.002	0.001
ESR	−0.594	−0.348	0.001	−0.920	−0.268
Cav-3	−0.382	−0.110	0.227	−1.005	0.241
Constant	69.368		<0.001	59.879	78.858

Cav-3, caveolin-3; ESR, erythrocyte sedimentation rate; NT-proBNP, N-terminal pro-brain natriuretic peptide; WBC, white blood cells.

### Serum Cav-3 by LVEF categories

Cav-3 concentrations across LVEF categories are detailed in Table 6. Median (IQR) Cav-3 was 4.81 (4.25–5.71) ng/L in Group 1 (HFrEF; LVEF < 40%, *n* = 28), 4.89 (4.40–5.58) ng/L in Group 2 (HFmrEF; LVEF 41%–49%, *n* = 17), and 3.98 (3.34–4.95) ng/L in Group 3 (LVEF > 50%, *n* = 45). Cav-3 was significantly higher in Group 1 vs. Group 3 (*p* = 0.004) and Group 2 vs. Group 3 (*p* = 0.008), with no difference between Group 1 and Group 2 (*p* = 0.855). This pattern indicates higher Cav-3 concentrations in both reduced and mildly reduced EF categories compared with individuals with preserved EF.

**Table 6:** Comparison of Cav-3 concentrations by LVEF.

Parameter	Cav-3 median (IQR1-IQR3)	*p*-value
Group 1: LVEF <%40 (*n* = 28)	4.81 (4.25–5.71)	**0.004** (Group 1 vs. Group 3)
Group 2: 41%≤LVEF<%49 (*n* = 17)	4.89 (4.40–5.58)	**0.008** (Group 2 vs. Group 3)
Group 3: LVEF >50% (*n* = 45)	3.98 (3.34–4.95)	0.855 (Group 1 vs. Group 2)

Bold indicates statistically significant values.

Cav-3, caveolin-3; LVEF, left ventricular ejection fraction.

## Discussion

In this case–control study, serum Cav-3 concentrations were higher in HF than in matched controls and showed the best discrimination when combined with NT-proBNP (AUC 0.878), exceeding either marker alone (NT-proBNP AUC 0.850; Cav-3 AUC 0.705). In multivariable analysis (WBC, NT-proBNP, D-dimer, ESR, Cav-3), EF was independently and negatively associated with WBC, NT-proBNP, and ESR, whereas Cav-3 did not retain significance after adjustment, suggesting complementary—rather than redundant—information from Cav-3 relative to inflammatory and neurohumoral markers.

Our biomarker profile aligns with prior literature showing that natriuretic peptides remain the strongest single analyte for HF discrimination and prognostication, yet are influenced by comorbid conditions such as obesity and chronic kidney disease, which can blunt or alter their levels and interpretability (13–15). Consistent with systemic activation in HF, we observed higher ESR, WBC, and D-dimer in HF; each has been linked to adverse outcomes in cardiovascular disease and HF cohorts, with D-dimer repeatedly associated with mortality and rehospitalization (16–18).

Beyond these routinely available markers, contemporary reviews emphasize a multimarker strategy integrating distinct biological axes—neurohormonal stress (NT-proBNP), inflammation/fibrosis (e.g., sST2, galectin-3), metabolism, endothelial biology, and remodeling—to improve risk classification (19–21). Our findings empirically support that approach: Cav-3 + NT-proBNP provided superior discrimination vs. NT-proBNP alone, indicating additive information rather than simple collinearity with natriuretic peptides or with inflammatory/coagulation markers (ESR, WBC, D-dimer).

Markers such as sST2 and galectin-3 capture mechano-inflammatory and profibrotic signaling and retain prognostic value even when natriuretic peptide levels are confounded by age/obesity; however, they primarily reflect downstream tissue response (20–22). In contrast, Cav-3 is a membrane microdomain scaffold that organizes β-adrenergic/L-type Ca^2+^-channel signaling within T-tubules; preclinical data show that perturbations of Cav-3/caveolae remodel excitation–contraction coupling and repolarization, and that targeted Cav-3 augmentation preserves microdomain structure and function (5, 23). This distinct, proximal position in cardiomyocyte signaling may explain why Cav-3 contributed orthogonal predictive signal to NT-proBNP and inflammatory/coagulation indices in our cohort.

Notably, while Cav-3 alone showed only moderate discrimination (AUC 0.705)—lower than NT-proBNP and comparable to ESR/D-dimer—its pairing with NT-proBNP yielded the highest AUC across all models we tested. This pattern mirrors recent HF biomarker frameworks advocating combined panels to capture complementary pathobiology rather than seeking a single “best” marker (19).

“Superiority” in contemporary risk models is typically judged incrementally (i.e., added AUC, NRI, calibration) over a clinical/biomarker baseline. In our data, Cav-3 was superior *in combination*—improving discrimination beyond NT-proBNP and outperforming other single inflammatory/coagulation markers when used as an augmenting biomarker. Two features may underlie this advantage: Mechanistic complementarity: Cav-3 indexes microdomain/T-tubule integrity and β-adrenergic–Ca^2+^ signaling architecture—axes not directly captured by natriuretic peptides, sST2, or galectin-3 (23). Potential resilience to common confounders: Given that NT-proBNP is depressed in obesity and altered by CKD/AF, a structurally anchored marker like Cav-3 could retain signal where peptides are noisier—an inference that warrants direct testing in enriched obese/CKD/AF subgroups (13, 14).

A recent PubMed-indexed scoping review on predictive biomarkers for HF highlights that combining markers from heterogeneous biological pathways improves early detection and risk stratification—especially those tied to endothelial biology and cellular remodeling (19). Our observation that Cav-3 + NT-proBNP outperforms NT-proBNP alone is consistent with this paradigm and suggests that membrane-microdomain biology is a useful addition to neurohormonal and inflammatory axes in composite scores.

Beyond risk prediction, increasing evidence links Cav-3/caveolae to HF-relevant pathophysiology, including arrhythmia susceptibility and insulin-signaling defects in ischemic or metabolic heart disease—both potential treatment targets (5, 24). Although therapeutic translation remains preclinical, these data strengthen the biological plausibility that a biomarker anchored in Cav-3 pathways might also be actionable, paralleling the trajectory seen with other microdomain-restoring strategies (23).

For routine practice, our results suggest that Cav-3 should not replace natriuretic peptides or established inflammatory markers; rather, it may augment them to refine discrimination and potentially identify phenotypes characterized by microdomain/T-tubule remodeling. If validated, a pragmatic panel could include NT-proBNP + Cav-3, with optional addition of ESR/D-dimer or sST2/galectin-3 depending on clinical context and assay availability (19–21).

Our single-center design and modest sample size limit precision, and Cav-3 lost significance in adjusted EF models—underscoring the need for larger, prospective studies with prespecified incremental-value metrics (*Δ*AUC, NRI, decision curves) vs. peptide-only and peptide + inflammatory baselines. Dedicated analyses in obesity, CKD, and AF cohorts are warranted to test whether Cav-3 is less susceptible to peptide confounding. Finally, head-to-head comparisons with sST2 and galectin-3 in the same cohort would clarify relative and additive utility.

Cav-3 captures a mechanistically distinct signal that enhances prediction when paired with NT-proBNP and relates to adverse remodeling, aligning with multi-marker HF strategies emphasized in recent PubMed-indexed reviews. Further prospective work should confirm incremental value and define the clinical scenarios in which Cav-3 proves superior to other markers by improving risk models rather than replacing existing standards (19).

At the study-derived cutoff, Cav-3 alone showed moderate accuracy (AUC 0.705; sensitivity 75.0%; specificity 73.3%), which we do not interpret as sufficient for a stand-alone test. Rather, our data support Cav-3 as an adjunctive marker: when combined with NT-proBNP, discrimination improved to an AUC of 0.878 with higher sensitivity (81.8%) and specificity (84.4%), exceeding either biomarker alone. In contemporary HF care, no single noninvasive analyte uniformly outperforms natriuretic peptides across all clinical contexts; therefore, the practical question is whether a candidate marker adds incremental information to existing models. Our results indicate that Cav-3 provides orthogonal signal to neurohormonal (NT-proBNP) and inflammatory/coagulation markers (ESR, WBC, D-dimer), consistent with its distinct mechanistic axis (T-tubule/caveolar microdomains).

Sensitivity and specificity also depend on the chosen threshold and intended use case (rule-out vs. rule-in). We selected a balanced cutoff (Youden index), which yields moderate values for each; alternative thresholds could prioritize sensitivity (screening) or specificity (confirmation) at the expected trade-off. Importantly, the clinical value of a biomarker is better established by incremental metrics—e.g., change in AUC, net reclassification improvement (NRI), calibration, and decision-curve analysis—relative to a peptide-based baseline. While our study was not powered for formal reclassification or net-benefit testing, these prospective analyses are planned and will clarify where Cav-3 is most effective (e.g., in populations where peptide interpretation is confounded by obesity, CKD, or AF).

In line with our results, the recent review by Licordari et al. (25) emphasized that the future of heart failure management lies in a precision medicine approach integrating non-natriuretic biomarkers with established markers such as NT-proBNP. They highlighted that emerging biomarkers reflecting inflammation, remodeling, metabolic stress, and oxidative injury add incremental value when used in multimarker strategies rather than as stand-alone tools (25). Our observation that Cav-3 improved diagnostic discrimination when combined with NT-proBNP is consistent with this paradigm, suggesting that Cav-3 represents a mechanistically distinct marker of microdomain/T-tubule remodeling that could enrich composite biomarker panels. By anchoring risk stratification in both neurohormonal stress and structural/microdomain biology, Cav-3 may contribute to the multidimensional patient phenotyping envisioned in contemporary precision medicine frameworks.

Taken together, we conclude that Cav-3 is not a replacement for NT-proBNP but a useful adjunct that measurably enhances diagnostic discrimination when used in combination. Larger, multicenter cohorts should validate incremental value and define clinically actionable thresholds for specific decision contexts.

## Conclusion

These data support Cav-3 as an adjunctive biomarker that can enhance diagnostic discrimination when added to NT-proBNP, rather than a replacement for current standards. Confirmation in larger, prospective, multicenter cohorts is needed to (i) validate reproducibility, (ii) establish actionable cut-offs, (iii) quantify incremental value over peptide-only and multi-marker baselines (e.g., ΔAUC, NRI, decision-curve analysis), (iv) compare head-to-head with sST2/galectin-3, and (v) test performance in obesity, CKD, and AF subgroups. Until such evidence is available, Cav-3 should be viewed as a promising, hypothesis-generating addition to multimarker HF assessment.

## Data Availability

The raw data supporting the conclusions of this article will be made available by the authors, without undue reservation.

## References

[1] Kieserman JM Myers VD Dubey P Cheung JY Feldman AM. Current landscape of heart failure gene therapy. J Am Heart Assoc. (2019) 8(10):e012239. 10.1161/JAHA.119.012239 31070089 PMC6585330

[2] Bhogal NK Hasan A Gorelik J. The development of compartmentation of cAMP signaling in cardiomyocytes: the role of T-tubules and caveolae microdomains. J Cardiovasc Dev Dis. (2018) 5(2):25. 10.3390/jcdd5020025 29751502 PMC6023514

[3] Kong CHT Bryant SM Watson JJ Roth DM Patel HH Cannell MB Cardiac-specific overexpression of caveolin-3 preserves t-tubular I_Ca_ during heart failure in mice. Exp Physiol. (2019) 104(5):654–66. 10.1113/EP087304 30786093 PMC6488395

[4] Horikawa YT Panneerselvam M Kawaraguchi Y Tsutsumi YM Ali SS Balijepalli RC Cardiac-specific overexpression of caveolin-3 attenuates cardiac hypertrophy and increases natriuretic peptide expression and signaling. J Am Coll Cardiol. (2011) 57(22):2273–83. 10.1016/j.jacc.2010.12.032 21616289 PMC3236642

[5] Markandeya YS Phelan LJ Woon MT Keefe AM Reynolds CR August BK Caveolin-3 overexpression attenuates cardiac hypertrophy via inhibition of T-type Ca2+ current modulated by protein kinase c*α* in cardiomyocytes. J Biol Chem. (2015) 290(36):22085–100. 10.1074/jbc.M115.674945 26170457 PMC4571960

[6] Tsutsumi YM Horikawa YT Jennings MM Kidd MW Niesman IR Yokoyama U Cardiac-specific overexpression of caveolin-3 induces endogenous cardiac protection by mimicking ischemic preconditioning. Circulation. (2008) 118(19):1979–88. 10.1161/CIRCULATIONAHA.108.788331 18936328 PMC2676165

[7] Meng Z Zhang Z Zhao J Liu C Yao P Zhang L Nitrative modification of caveolin-3: a novel mechanism of cardiac insulin resistance and a potential therapeutic target against ischemic heart failure in prediabetic animals. Circulation. (2023) 147(15):1162–79. 10.1161/CIRCULATIONAHA.122.063073 36883479 PMC10085855

[8] Li J Richmond B Cluntun AA Bia R Walsh MA Shaw K Cardiac gene therapy treats diabetic cardiomyopathy and lowers blood glucose. JCI Insight. (2023) 8(18):e166713. 10.1172/jci.insight.166713 37639557 PMC10561727

[9] Khan MS Smego D Li J Ishidoya Y Offei E Ruiz Castillo MS AAV9-cBIN1 gene therapy rescues chronic heart failure due to ischemic cardiomyopathy in a canine model. Commun Med (Lond). (2025) 5(1):93. 10.1038/s43856-025-00787-w 40148575 PMC11950290

[10] Tian J Popal MS Huang R Zhang M Zhao X Zhang M Caveolin as a novel potential therapeutic target in cardiac and vascular diseases. A mini review. Aging Dis. (2020) 11(2):378–89. 10.14336/AD.2019.09603 32257548 PMC7069461

[11] Sellers SL Trane AE Bernatchez PN. Caveolin as a potential drug target for cardiovascular protection. Front Physiol. (2012) 3:280. 10.3389/fphys.2012.00280 22934034 PMC3429054

[12] Sun LY Qu X Chen LZ Zheng GS Wu XL Chen XX Potential roles of serum caveolin-3 levels in patients with atrial fibrillation. Front Aging Neurosci. (2017) 9:90. 10.3389/fnagi.2017.00090 28420984 PMC5378709

[13] Madamanchi C Alhosaini H Sumida A Runge MS. Obesity and natriuretic peptides, BNP and NT-proBNP: mechanisms and diagnostic implications for heart failure. Int J Cardiol. (2014) 176(3):611–7. 10.1016/j.ijcard.2014.08.007 25156856 PMC4201035

[14] Sasaki T Oishi E Nagata T Sakata S Chen S Furuta Y N-Terminal pro-B-type natriuretic peptide and incident CKD. Kidney Int Rep. (2021) 6(4):976–85. 10.1016/j.ekir.2021.01.006 33912747 PMC8071624

[15] Vergaro G Gentile F Meems LMG Aimo A Januzzi JL Jr Richards AM NT-proBNP for risk prediction in heart failure: identification of optimal cutoffs across body mass index categories. JACC Heart Fail. (2021) 9(9):653–63. 10.1016/j.jchf.2021.05.014 34246607

[16] Huang B Li YJ Shen J Yang Y Liu G Luo SX. D-dimer level and long-term outcome in patients with end-stage heart failure secondary to idiopathic dilated cardiomyopathy. J Geriatr Cardiol. (2019) 16(8):621–9. 10.11909/j.issn.1671-5411.2019.08.005 31555330 PMC6748897

[17] Huang Y Yang LH Li YX Chen H Li JH Su HB The value of D-dimer in the prognosis of dilated cardiomyopathy: a retrospective cohort study. Sci Rep. (2024) 14(1):26806. 10.1038/s41598-024-76716-z 39500987 PMC11538493

[18] Simes J Robledo KP White HD Espinoza D Stewart RA Sullivan DR D-dimer predicts long-term cause-specific mortality, cardiovascular events, and cancer in patients with stable coronary heart disease: lIPID study. Circulation. (2018) 138(7):712–23. 10.1161/CIRCULATIONAHA.117.029901 29367425

[19] Mariappan V Srinivasan R Pratheesh R Jujjuvarapu MR Pillai AB. Predictive biomarkers for the early detection and management of heart failure. Heart Fail Rev. (2024) 29(2):331–53. 10.1007/s10741-023-10347-w 37702877

[20] Riccardi M Myhre PL Zelniker TA Metra M Januzzi JL Inciardi RM. Soluble ST2 in heart failure: a clinical role beyond B-type natriuretic peptide. J Cardiovasc Dev Dis. (2023) 10(11):468. 10.3390/jcdd10110468 37998526 PMC10672197

[21] Martuszewski A Paluszkiewicz P Poręba R Gać P. Galectin-3 in cardiovascular health: a narrative review based on life’s essential 8 and life’s simple 7 frameworks. Curr Issues Mol Biol. (2025) 47(5):332. 10.3390/cimb47050332 40699731 PMC12110310

[22] Vergaro G Aimo A Januzzi JL Jr Richards AM Lam CSP Latini R Cardiac biomarkers retain prognostic significance in patients with heart failure and chronic obstructive pulmonary disease. J Cardiovasc Med (Hagerstown). (2022) 23(1):28–36. 10.2459/JCM.0000000000001281 34839321

[23] An Z Tian J Zhao X Liu L Yang X Zhang M Regulation of cardiovascular and cardiac functions by caveolins. FEBS J. (2024) 291(17):3753–61. 10.1111/febs.16798 37060249

[24] Sadoshima J. Caveolin-3 nitration drives insulin resistance in prediabetic hearts. Circulation. (2023) 147(15):1180–2. 10.1161/CIRCULATIONAHA.123.064250 37036910 PMC10091234

[25] Licordari R Correale M Bonanno S Beltrami M Ciccarelli M Micari A Beyond natriuretic peptides: unveiling the power of emerging biomarkers in heart failure. Biomolecules. (2024) 14(3):309. 10.3390/biom14030309 38540729 PMC10967756

